# Decommissioning of Durable Left Ventricular Assist Devices

**DOI:** 10.1016/j.cjco.2026.04.017

**Published:** 2026-05-07

**Authors:** Ryaan EL-Andari, Holger Buchholz, Jennifer Conway, Tara Pidborochynski, Darren H. Freed, Steven R. Meyer, Roderick G.G. MacArthur

**Affiliations:** a Division of Cardiac Surgery, University of Alberta, Edmonton, Alberta, Canada; b Division of Pediatric Cardiology, Stollery Children's Hospital, Edmonton, Alberta, Canada; c Division of Pediatrics, Stollery Children's Hospital, Edmonton, Alberta, Canada; d Division of Congenital Cardiac Surgery, University of Alberta, Edmonton, Alberta, Canada

**Keywords:** ventricular assist device, left ventricular assist device, decommission

## Abstract

Cardiac recovery following durable left ventricular assist device (LVAD) implantation is the ideal outcome for heart failure patients. Traditionally, the durable LVAD has required explantation. We describe our single-centre experience with LVAD decommissioning. LVAD decommissioning involved making a sternotomy or sub-xiphoid incision, ligating the outflow graft, and removing the driveline by transecting the driveline inside and outside of the chest. Six patients underwent durable LVAD decommissioning from 2017-2024. One patient had a HeartWare (Medtronic, Galway, Ireland) device, 3 patients had a HeartMate II (Abbott, Abbott Park, IL), and 2 patients had a HeartMate 3 (Abbott). All patients were successfully decommissioned and discharged without mechanical circulatory support.

## Résumé

La récupération cardiaque après la pose d'un dispositif d'assistance ventriculaire gauche (DAVG) de longue durée constitue l'issue idéale pour les patients atteints d'insuffisance cardiaque. Traditionnellement, le DAVG de longue durée nécessitait une explantation. Nous décrivons notre expérience monocentrique en matière de retrait de DAVG. Ce retrait consistait à pratiquer une sternotomie ou une incision sous-xiphoïdienne, à ligaturer le greffon de sortie et à retirer le câble percutané en le sectionnant à l'intérieur et à l'extérieur du thorax. Six patients ont subi un retrait de DAVG de longue durée entre 2017 et 2024. Un patient était équipé d'un dispositif HeartWare, trois patients d'un HeartMate II et deux patients d'un HeartMate 3. Tous les patients ont été déconnectés avec succès et ont pu sortir de l'hôpital sans assistance circulatoire mécanique.

Durable left ventricular (LV) assist devices (LVADs) have been utilized increasingly in recent years for patients with end-stage heart failure.^1^^,^^2^ Durable LVADs are most often used as a bridge to transplantation or for destination therapy in patients who are not eligible or not amenable to heart transplantation. In some cases, a durable LVAD can be used as a bridge to recovery—the cardiac function recovers such that the LVAD is no longer required and can be removed.^2^, ^3^, ^4^

The traditional approach to discontinuation of durable LVAD therapy has been to excise the LVAD with all of its components. This intervention carries significant risk associated with an invasive procedure, prolonged operative times, and left ventricular repair, which also carries risk of thrombosis at the site of repair or plug. A newer approach to durable LVAD discontinuation, called “decommissioning,” has been utilized increasingly.

The traditional approach to decommissioning of a durable LVAD involves ligation of the outflow graft and removal of the driveline. The LVAD itself is left in place, and the heart is able to function without LVAD support but with the device still in-situ. A more recently described approach is that of percutaneous decommissioning, which involves deployment of vascular plug devices to occlude the outflow graft, and cutting of the driveline.^3^^,^^5^ Minimally invasive approaches to LVAD explantation also have been described using smaller thoracotomies to access the LVAD.^6^ LVAD decommissioning has been described with older versions of durable LVADs, although very few cases have been reported in the literature for newer generations such as the HeartMate 3 (HM3; Abbott, Abbott Park, IL), and with varying approaches.^1^^,^^3^^,^^4^^,^^7^ Herein, we describe our single-centre experience with LVAD decommissioning, surgical technique, and postoperative outcomes.

## Methods

### Ethics

The local research ethics board provided approval for this study under Pro00142257 on August 28, 2024, with an individual waiver for consent.

### Patients

All patients who underwent durable LVAD implantation at the Mazankowski Alberta Heart Institute, followed by recovery of cardiac function and subsequent decommissioning of the LVAD, from January 2017 to April 2024, were included in this study. All durable LVAD types were included. Baseline demographic data from patients were collected, including age, sex, etiology of heart failure, type of durable LVAD, and preoperative comorbidities.

### Outcomes

The primary outcome was all-cause mortality at longest follow-up. Secondary outcomes included rates of infection, recurrent heart failure, reoperation, heart transplant, cerebrovascular accident, and acute kidney injury. The last day of the follow-up period was December 31, 2024.

### Preoperative weaning protocol

The preoperative weaning protocol to assess for heart function prior to decommissioning involves identification of improvement of cardiac function on echocardiography and a right heart catheterization. The patient is brought to the catheterization laboratory, where a pulmonary artery catheter is placed. Measurements of heart function, including right atrial pressure, right ventricular pressure, pulmonary artery pressure, pulmonary capillary wedge pressure (PCWP), and cardiac output, are taken at the baseline LVAD revolutions per minute (rpm) speed. The LVAD is then decreased to a neutral speed —approximately 8000 rpm for the HeartMate II (Abbott) and 4000 rpm for the HM3. Measurements are then retaken at 2 minutes and 15 minutes after LVAD speed is set to neutral. The patient is then taken from the catheterization laboratory at neutral LVAD rpms and given a 6-minute walk test. Then the patient is taken back to the catheterization laboratory to repeat the pulmonary artery catheter measures. The test is then complete, and the LVAD is returned to the baseline speeds.

Target parameters to use to consider decommissioning include the following: a left ventricular ejection fraction (LVEF) > 45%; left ventricular end diastolic diameter < 60 mm; left ventricular end systolic diameter < 50 mm; peak oxygen consumption (VO2) > 16 mL/kg/min and > 50% predicted; VE/VCO2 slope < 34 (minute ventilation/carbon dioxide production), cardiac index > 2.2 L/min; PCWP < 15 mm Hg and post 6-minute walk test cardiac index > 2.2 L/min; and PCWP < 18 mm Hg.

### Surgical technique

**Figure 1:**
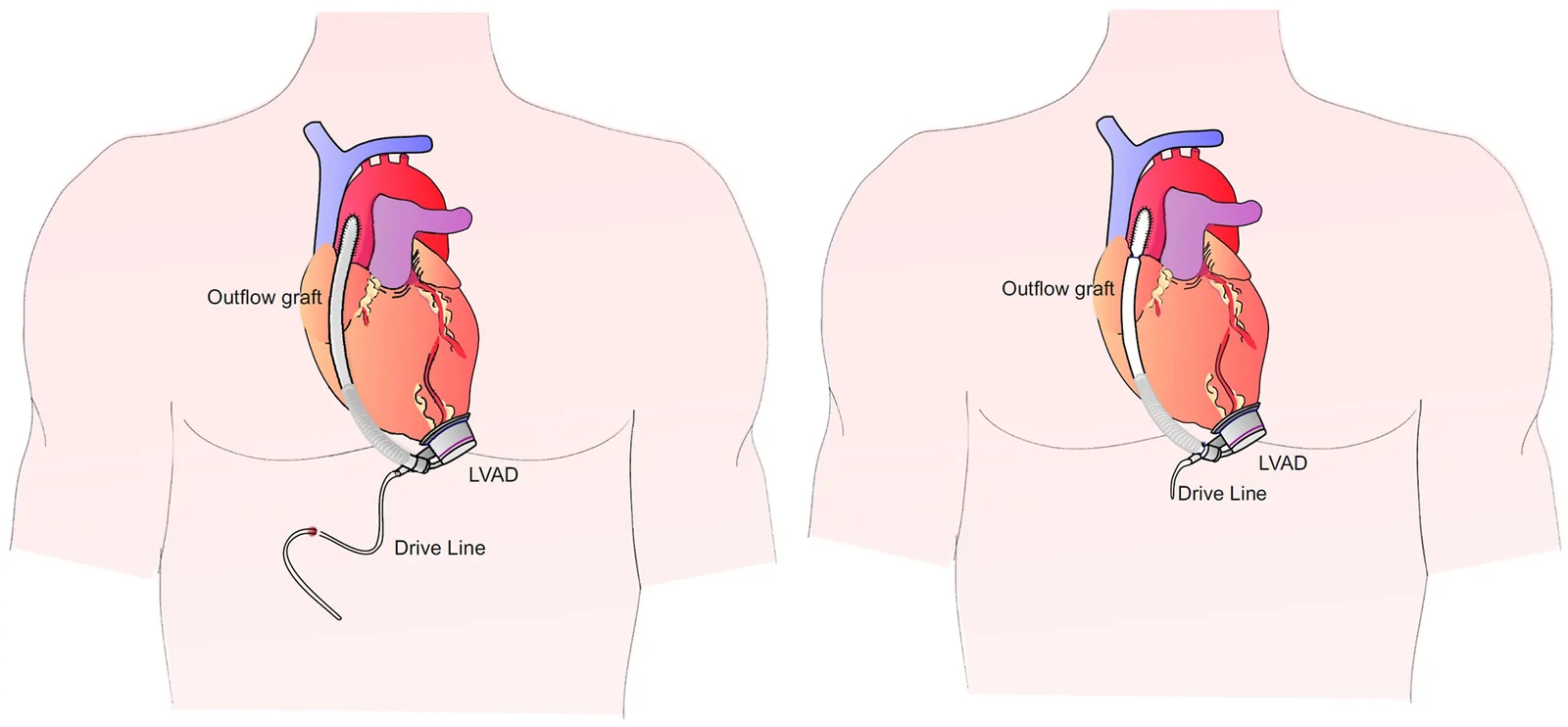
Illustration of the decommissioning of a left ventricular assist device with removal of the driveline and ligation of the outflow graft.

The patients are brought to the operating room where they are prepped and draped. An incision is made over the lower sternum, or xiphoid sternum, or a complete redo sternotomy is performed. For the lower sternotomy, the xiphoid sternum is either cut or dissected out and resected to allow for adequate visualization. A blade is used to carefully open the bend relief to reveal the dacron outflow graft within. Two heavy silk sutures are placed around the outflow graft in preparation for ligation. The intraoperative weaning protocol includes turning down the LVAD rpms and eventually clamping the outflow graft for at least 15 minutes, ensuring hemodynamic stability throughout, prior to decommissioning. The LVAD is then discontinued, the pump and housing left in place, and the outflow graft is tied off (Fig. 1). The bend relief is then closed over the outflow graft with a running 4-0 Prolene suture (Ethicon, Raritan, NJ).

The driveline is then dissected within the substernal area to the diaphragmatic surface of the pericardium. The driveline is then transected and dissected distally within the abdominal wall, and it is cut once again (Fig. 1). The upper abdomen and lower sternal area are closed in standard fashion. A button of skin is then cut around the driveline and it is dissected out further and ultimately removed. The wound is then gently irrigated, and the incision is closed with polypropylene sutures. Postoperatively, all patients are anticoagulated with unfractionated heparin and transitioned to warfarin with an international normalized ratio target of 2-3 or a direct oral anticoagulant for a duration of at least 1 year when possible. Post-decommissioning medical therapy includes heart failure guideline-directed medical therapy as tolerated, including angiotensin-converting enzyme (ACE) inhibitors or angiotensin receptor blockers, beta-blockers, potassium-sparing diuretics, and sodium-glucose cotransporter 2 inhibitors.

Similar techniques have been described in other settings with previous generations of LVADs, such as the HMII and HeartWare (Medtronic, Galway, Ireland) devices, with some technical differences, including transection and oversewing of the graft or using a surgical stapler to occlude the outflow graft in some reports.^8^, ^9^, ^10^

## Results

### Baseline demographics

Six patients underwent durable LVAD decommissioning from 2017-2024 (Table 1). During this time, 294 durable LVADs were implanted at our centre. Five patients were male and one was female. The median age of the included patients was 41.2 years (interquartile range [IQR] 28.4-50.7). The etiology of the heart failure was dilated idiopathic cardiomyopathy in 3 cases, spontaneous coronary artery dissection postpartum resulting in myocardial infarction in 1 case, ischemic cardiomyopathy in 1 case, and valvular heart disease with postoperative heart failure in 1 case. In 5 cases, the LVEF was < 15%, and 1 patient had a pre-LVAD implantation LVEF of 30%-35% due to hemodynamic instability following myocardial infarction requiring temporary LVAD that could not be weaned. One patient received a HeartWare device, 3 received a HMII, and 2 received a HM3. At the time of LVAD decommissioning, one patient had slightly improved heart function from an LVEF of 30%-35% to 35%-40%, although this patient passed several weaning trials preceding LVAD decommissioning. The remaining 5 patients had an increase from < 15% to ≥ 50% LVEF. The median time to decommissioning was 477 days (IQR 399-641).

**Table 1:** Characteristics and outcomes of the included patients undergoing durable left ventricular assist device (LVAD) decommissioning

Baseline demographics	Patient 1	Patient 2	Patient 3	Patient 4	Patient 5	Patient 6
Age at the time of durable LVAD implantation, Y	26	52	32	23	51	49
Sex	Male	Male	Female	Male	Male	Male
Comorbidities	Pulmonary disease, ventricular arrhythmia, substance abuse	Pulmonary disease, pulmonary hypertension, substance abuse	Frailty	None	Atrial fibrillation	Pulmonary hypertension
Etiology of heart failure	DCM	DCM	Postpartum cardiomyopathy	DCM	Ischemic cardiomyopathy	VHD, postoperative HF
**Echocardiographic data prior to LVAD implantation**						
LVEF at time of durable LVAD implantation	10%	10%	30%–35%	< 20%	< 20%	< 20%
LVEDd, cm	6.6	6.8	—	8.5	6.2	7.1
LVESd, cm	6.1	6.4	—	8.2	5.5	6.5
Right ventricular systolic pressure, mm Hg	—	—	—	68.5	53.0	34.0
**LVAD details**						
Preoperative temporary MCS	Temporary LVAD	N/A	Temporary LVAD	N/A	N/A	N/A
Duration of preoperative temporary MCS	36 d	N/A	3 d	N/A	N/A	N/A
Durable LVAD	HeartMate II	HeartMate II	HeartWare	HeartMate II	HeartMate 3	HeartMate 3
Time to decommissioning, d	553	713	204	670	400	399
Surgical approach	Sternotomy	Sternotomy	Hemi sternotomy	Hemi sternotomy	Sub-xiphoid	Hemi sternotomy
Post-decommissioning anticoagulation strategy	Warfarin: INR target 2–3	Warfarin: INR target 1.5–2	Warfarin: INR target 2–2.5, discontinued after 1.5 Y	Warfarin: INR target 2–3, discontinued after 9 mo	Warfarin: INR target 2–3, discontinued after 10 mo	Warfarin: INR target 2–3 for 6 mo, DOAC: 6 mo
**Decommissioning parameters**						
LVEF (> 45%)	—	55	35	50	55	50
Cardiac index (> 2.2 l pm)	—	2.3	2.7	2.5	2.2	2.3
PCWP (< 15 mm Hg)	—	6	16	7	10	11
**Post 6-minute walk test**						
Cardiac index (> 2.2 l pm)	—	2.6	2.9	2.7	2.2	2.2
PCWP (< 18 mm Hg)	—	5	14	15	14	17
**Outcomes**						
Hospital length of stay, d	7	16	22	10	6	18
Follow-up time, mo	95	85	81	62	31	9
Mortality during follow-up period	No	No	No	No	No	No
Readmission for HF	No	No	2 mo post-discharge	No	No	No
Infection	No	No	No	Yes	No	No
Myocardial infarction	No	No	No	No	No	No
CVA	No	No	No	No	No	No
Heart transplantation	No	No	No	No	No	No
Reoperation	LVAD explantation at 58 mo	No	Mediastinal exploration POD 2	Yes	No	No

HeartMate II and HeartMate3 (Abbott, Abbott Park, IL); HeartWare (Medtronic, Galway, Ireland).

CVA, cerebrovascular accident; DCM, dilated cardiomyopathy; DOAC, direct oral anticoagulant; HF, heart failure; ICM, ischemic cardiomyopathy; INR, international normalized ratio; LVEDd, left ventricular end diastolic diameter; LVEF, left ventricular ejection fraction; LVESd, left ventricular end systolic diameter; MCS, mechanical circulatory support; N/A, not applicable; PCWP, pulmonary capillary wedge pressure; POD, postoperative day; VHD, valvular heart disease.

The dashes represent data that was not available for the individual patient.

### Outcomes

The median hospital length of stay post-decommissioning was 13 days (IQR 7.8-17.5). The median follow-up time was 71.5 months (IQR 38.8-84). During the follow-up period, 1 patient (patient 3) required reoperation on postoperative day 2 for mediastinal exploration, as a clot was identified on echocardiogram that appeared to be compressing the right ventricle. Following exploration, no hemodynamically significant collection was identified. Patient 3 was readmitted to the hospital 2 months post-LVAD decommissioning, for symptoms of fatigue, and underwent a heart failure workup. Workup revealed an ejection fraction of 40%-45% on echocardiogram, and right heart catheterization showed a PCWP of 21 mm Hg, a transpulmonary gradient of 6 mm Hg, and a cardiac index of 2.28. The total hospital length of stay was 4 days. Another patient experienced recurrent infections of the pump that required explant at 58 months post-decommissioning, owing to a chronically draining fistula that became infected and progressed to the LVAD. No mortality, myocardial infarction, cerebrovascular accidents, or heart transplantation occurred during the follow-up period.

### Follow-up echocardiographic data

Follow-up echocardiograms were available for 5 of 6 patients between 0.6-3 years post–LVAD decommissioning. LVEF ranged from 40%-60% in all patients. No hemodynamically significant valve lesions occurred, and right ventricular systolic pressure was in the range of 23-41 mm Hg (Table 2; Supplemental Fig. S1).

**Table 2:** Echocardiographic results following left ventricular assist devicedecommissioning.

Echocardiogram	Patient 1	Patient 2	Patient 3	Patient 4	Patient 5	Patient 6
**Echocardiogram 1**						
Time to echocardiogram, Y	—	2	3	1	1	0.6
LVEF, %	—	43	47	55–60	40	45–50
Aortic insufficiency	—	Trace	None	None	None	Mild
Mitral regurgitation	—	Moderate	Mild	Trace	Trace	Mild
Tricuspid regurgitation	—	Trace	Mild	Mild	Mild	Mild
RVSP, mm Hg	—	29	—	23	41	—
**Echocardiogram 2**						
Time to echocardiogram, Y	—	5	5	5	—	—
LVEF, %	—	38	55	46	—	—
Aortic insufficiency	—	None	None	None	—	—
Mitral regurgitation	—	Mild—moderate	Mild	Trace	—	—
Tricuspid regurgitation	—	Trace	Mild	Mild	—	—
RVSP, mm Hg	—	—	26	26	—	—

LVEF, left ventricular ejection fraction; RVSP, right ventricular systolic pressure.

## Discussion

In this case series, we describe a series of durable LVAD decommissioning, including the HMII and HM3 LVADs, with a follow-up period to 95 months. The description of this cohort provides a valuable addition to the literature regarding the management options of durable LVAD patients with recovery of cardiac function. Durable LVADs are used most commonly as a bridge to transplantation or destination therapy. Cardiac recovery, while it is the ideal outcome, is uncommon and reported to occur in only approximately 1% of patients with a durable^2^^,^^11^ LVAD.

Although this number historically has been low, the number of durable LVAD implantations is growing.^4^^,^^5^ This increase will inevitably result in increasing numbers of LVAD patients who achieve recovery of cardiac function adequate to allow for deactivation of the LVAD. The standard approach to these cases has long been the complete removal of the LVAD device and its associated hardware. At our centre, LVAD explant was previously the method utilized, with approximately 5 LVAD explants performed to date, as the initial approach to cardiac recovery. However, decommissioning has become the preferred method in recent years, and its incidence has surpassed the total number of LVAD explants. Explantation is associated with increased risk, given the difficulty associated with reoperation and removal of all LVAD components. Explantation also adds an additional insult to the heart, requiring extensive surgery and cardiopulmonary bypass. Important to note is that the LV apical cannulation site requires repair with LVAD explantation. A simple patch of the LV apex results in a defect in the endocardial wall that will increase the likelihood of thrombus formation and bleeding at the patch site, and the availability of dedicated apical cannulation site plugs is limited.^12^ Due to these limitations of complete explant of LVAD devices, decommissioning has become a more frequently utilized procedure at our centre. Patients who present with recovery of heart function, and who no longer require LVAD support, but do not have a strong indication for complete explantation, such as infection, are now referred for LVAD decommissioning. The patients included in this cohort were relatively young, necessitating a balance between short-term risk and acceptable long-term outcomes. Only limited long-term data are available on the outcomes of patients post-LVAD decommissioning, especially with newer LVAD devices. The unknown long-term outcomes of LVAD decommissioning must be balanced against the risks of complete explantation, including a more invasive surgical procedure and risk of apical thrombosis.

LVAD explantation has been the traditional approach to terminating LVAD support in patients with recovery of cardiac function. Several series on patients who have undergone LVAD explantation have been published previously. Potapov and colleagues reported the outcomes of 68 patients who underwent LVAD explantation from 54 centres in Europe.^12^ The 1-year survival of this cohort was 90%, with a 5-year survival of 80%, however, the median follow-up time was 34 months. In this cohort, 7% of patients required reimplantation of an LVAD, 3% underwent heart transplantation, and 31% were in New York Heart Association class III or IV during the follow-up period. Another European study reported outcomes from EUROMACS (European Registry for Patients with Mechanical Circulatory Support) including 45 patients.^2^ Follow-up data after explantation were only available for 28 patients, with 1 death (3.6%), 3 heart failure recurrences (10.7%), and 1 LVAD reimplantation (3.6%) at a median follow-up time of 26 months. In comparison, outcomes of LVAD decommissioning generally have been positive. Gerhard and colleagues reported a 2-centre experience with 17 patients undergoing surgical LVAD decommissioning.^13^ One patient had recurrent heart failure (6%), 3 had recurrent infections resulting in death (18%), and 1 patient required LVAD explantation for infections (6%). Of note, 3 patients (18%) experienced thrombotic complications, but these were all in HVAD devices. MacGowan et al. reported the outcomes of 9 patients who underwent surgical LVAD decommissioning with 2 deaths at 413 and 810 days post-decommissioning.^9^ Finally, a systematic review by Choi et al. compared the outcomes of LVAD explantation and decommissioning, which included 85 patients. This review found no significant differences between approaches in terms of mortality, cerebrovascular accidents, infection, or heart failure.^4^ However, a trend was present toward increased rates of heart failure in patients who underwent decommissioning. Percutaneous approaches to decommissioning also have been reported. Abdallah and colleagues reported the outcomes of 6 patients post-percutaneous decommissioning, with no mortality or adverse events within a mean follow-up period of 18 months.^5^ Moroni et al. reported the outcomes of 5 patients who underwent percutaneous decommissioning, with 1 mortality (20%) and 1 patient having recurrent heart failure requiring a total artificial heart (20%).^3^ Moroni et al, also summarized the available literature on the topic with case reports and small series, generally reporting positive outcomes, but with limited numbers. Of the 20 reported cases, with almost all having ≤ 2 years of follow-up care, 3 patients (15%) required heart transplantation, 2 died (10%), and the remainder were alive, with 1 lost to follow-up and 1 in palliative care at the time of publication.

The decommissioning approach to LVAD deactivation provides several benefits. This technique allows for a minimally invasive approach through a lower hemisternotomy, subcostal incision, thoracotomy, or even hybrid surgical and percutaneous decommissioning.^4^^,^^5^^,^^14^^,^^15^ Additionally, leaving the LVAD in place obviates the need for an LV apical repair. Finally, this approach allows for a reduced upfront surgical risk by reducing the operative times and invasiveness of the procedure. Of note, a significant limitation of this approach is the risk of thrombus formation at the outflow or inflow cannulation sites, due to stasis.^3^^,^^4^ The strategy followed at our centre utilizes heparin in the intensive care unit setting with planned anticoagulation for 1 year postoperatively to allow for endothelialization of the inflow and outflow sites and for prevention of thrombus formation in the long term. Although consensus on anticoagulation strategies post–LVAD decommissioning has yet to be reached, this strategy is in line with other reports in the literature.^14^ Another risk associated with LVAD decommissioning is the retention of hardware, which is at risk of infection, a result that occurred in 1 of the patients described in this cohort. Patients with a previously diagnosed pump infection should be considered for LVAD explantation instead of decommissioning, given the increased risk of recurrent or wosening infection.

### Limitations

The sample size of this study is small. Although the longest follow-up period of this study was 95 months, not all patients were followed for this long, and future follow-up evaluation is necessary to elucidate the long-term outcomes of these patients.

## Conclusion

Of the outcome options following durable LVAD implantation, heart function recovery is the most sought-after and least common outcome. Following cardiac recovery, LVAD decommissioning allows for a less invasive approach to cessation of LVAD therapy. As demonstrated in this case series, LVAD decommissioning is safe, feasible with current durable LVADs, can be performed with minimal invasiveness, and allows for long-term survival following cardiac recovery.

## Data Availability

The data underlying this article are not permitted to be shared publicly.

## Ethics Statement

The local research ethics board approved this study under Pro00142257 on August 28, 2024 with individual waiver for consent and adheres to the CARE guidelines.

## Patient Consent

The authors confirm that patient consent is not applicable to this article. The local research ethics board approved this study under Pro00142257 on August 28, 2024 with individual waiver for consent.

## Funding Sources

The authors have no funding sources to declare.

## Disclosures

J.C. reports receiving an unrestricted education grant from Abbott, medical monitor of the Pumps for Kids, Infants, and Neonates (PumpKIN) Trial. H.B. reports receiving consulting fees from Abbott. The other authors have no conflicts of interest to disclose.
