# Supplementary material for: Decommissioning of Durable Left Ventricular Assist Devices
Source: CJC Open. 2026 May 7;8(9):1177–82. doi: 10.1016/j.cjco.2026.04.017 (PMC13583574; doi:10.1016/j.cjco.2026.04.017)

Decommissioning of Durable Left Ventricular Assist Devices

Supplemental Materials

Ryaan EL-Andari[1], MD., Holger Buchholz[1], MD., Jennifer Conway[2], MD, MSc., Tara Pidborochynski[3], MSc., Darren H. Freed[4], MD, PhD., Steven R. Meyer[1], MD, PhD., Roderick GG MacArthur[1], MD, MSc.

[1]Division of Cardiac Surgery, University of Alberta, Edmonton, Alberta, Canada.
[2]Division of Pediatric Cardiology, Stollery Children's Hospital, Edmonton, AB, Canada.
[3]Division of Pediatrics, Stollery Children's Hospital, Edmonton, AB, Canada.
[4]Division of Congenital Cardiac Surgery, University of Alberta, Edmonton, Alberta, Canada.

Running Title: Durable LVAD Decommissioning
Funding: None
Declarations of Interest: None
Keywords: Ventricular assist device, LVAD, decommission.
Word count: 165

Corresponding Author:
Ryaan EL-Andari, MD
University of Alberta
Edmonton, Alberta, Canada
Email:elandari@ualberta.ca

Post-publication Corresponding Author:
Roderick MacArthur, MD, MSc, FRCSC
Director of Cardiac Surgery
Clinical Professor of Surgery
University of Alberta

Mazankowski Alberta Heart Institute
11220 83 Ave NW, Edmonton, AB
p:1-780-407-2186
Email: roderick.macarthur@ahs.ca

41 **Supplemental Figure Legends**

42 Supplemental Figure S1. Graph showing the trends in left ventricular ejection fraction for patients 2-6
43 after left ventricular assist device decommissioning.

44 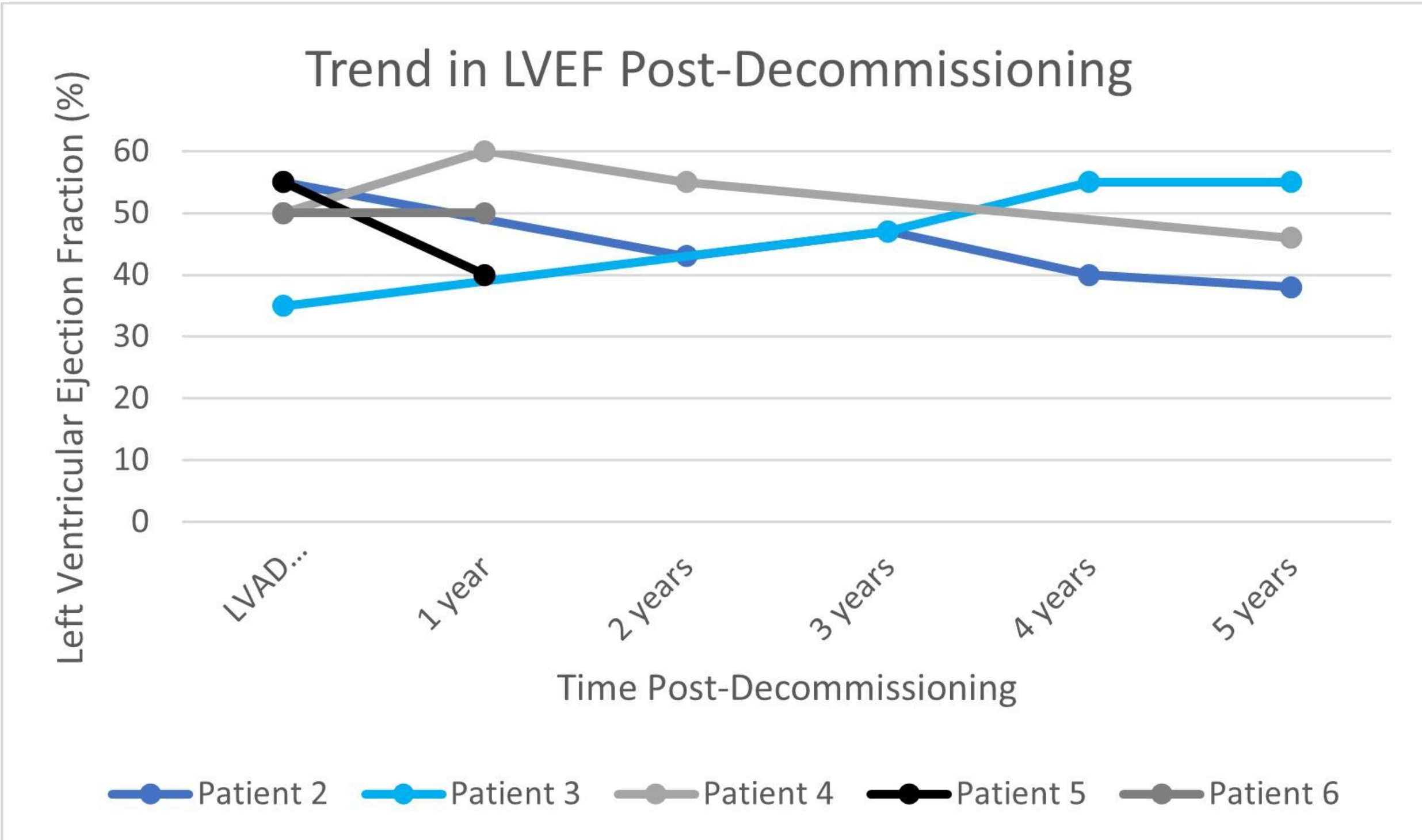

Supplement: Supplementary file 1 [file mmc1.pdf]
